# Genetic variations in *GJA3, GJA8*, *LIM2*, and age-related cataract in the Chinese population: a mutation screening study

**Published:** 2011-02-26

**Authors:** Zhou Zhou, Binbin Wang, Shanshan Hu, Chunmei Zhang, Xu Ma, Yanhua Qi

**Affiliations:** 1 Department of Ophthalmology, the Second Affiliated Hospital of Harbin Medical University, Harbin, China; 2 Department of Genetics, National Research Institute for Family Planning, Beijing, China

## Abstract

**Purpose:**

To investigate the role of genetic variations in three known cataract-associated genes, gap junction protein α3 (*GJA3*), gap junction protein α8 (*GJA8*), lens intrinsic membrane protein 2 (*LIM2*), encoding lens fiber cell membrane proteins in the development of age-related cataracts.

**Methods:**

One hundred and forty-five sporadic age-related cataract patients and one hundred and fifty-six unrelated random healthy controls participated in this study. Genomic DNA was extracted from peripheral blood leukocytes. All exons of *GJA3*, *GJA8*, and *LIM2* were sequenced after being amplified by polymerase chain reaction (PCR). The functional consequences of the mutations were analyzed using PolyPhen.

**Results:**

We found five novel variations in 145 patients and none of them presented in the 156 controls. There are two variations in *GJA3* (c.-39C>G, c. 415G>A); one in *GJA8* (c. 823G>A), and two in *LIM2* (c.57G>A, c.67A>C). PolyPhen predicted that the *LIM2* c.67A>C mutation may have potential pathogenicity.

**Conclusions:**

The genetic mutation in *GJA3*, *GJA8*, and *LIM2* may slightly contribute to the development of age-related cataracts. This study showed a potential relationship between lens fiber cell membrane protein genes and the development of age-related cataracts in the Chinese population.

## Introduction

Age-related cataract (ARC) is the leading cause of low vision and blindness in Asia [1,2]. As the world’s population ages, cataract-induced visual dysfunction and blindness is on the increase [3]. Several studies have identified the risk factors for cataract, including being of the female sex, having lower socioeconomic status, having diabetes mellitus, smoking, and lower body mass index [4-6]. Recently, some twin studies provided evidence for the contribution of genetic factors in the pathogenesis of ARC. In 2000 and 2001, Hammond et al. recognized that the heritability for age-related cortical cataract was 53%–58% [7] and approximately 48% for nuclear cataract [8].

In the past few years, attempts have been made to identify new loci for ARC. Until now, at least nine genes associated with congenital cataract were proven to link to ARC, including EPH receptor A2 (*EPHA2*, 1p) [9], gap junction protein α8 (*GJA8*, 1q) [10], galactose-1-phosphate uridylyltransferase (*GALT*, 9p) [11], solute carrier family 16, member 12 (monocarboxylic acid transporter 12; *SLC16A12*, 10q)  [12], heat shock transcription factor 4 (*HSF4*, 16q) [13], galactokinase 1 (*GALK1*, 17q) [14], ferritin light polypeptide (*FTL*, 19q) [15], crystallin αA (*CRYAA*, 21q) [16], and  crystallin βB2 (*CRYBB2*, 22q) [17]. However, the genes associated clearly with adult-onset cataract are still few compared to those with congenital cataract. There was a theory that mutations which severely disrupted the lens cell architecture or environment might produce congenital cataract, while other relatively mild mutations might contribute to age-related cataract [18]. According to this hypothesis we selected some candidate genes from those associated with congenital cataract as our objects. Gap junction protein α3 (*GJA3*), gap junction protein α8 (*GJA8*), and lens intrinsic membrane protein 2 (*LIM2*) are genes that encode proteins on the lens fiber cell membrane. These proteins play a decisive role in the growth and differentiation of lens fiber cells as well as in the maintenance of eye lens transparency.

In this work, we screened all exons and the flanking sequences of *GJA3*, *GJA8*, and *LIM2* in a total of 301 case-control individuals and found some novel mutations. Meanwhile, some of the mutations may have potential pathogenicity, since they may disrupt the process of transcription or translation or the normal function of the translation product.

## Methods

### Patients and controls

One hundred and forty-five patients with age-related cataracts were collected during our clinical work. Cataract diagnosis was determined according to lens opacities classification system III (LOCSIII) [19]. All patients had bilateral cataracts and the severity of cortical or nuclear cataracts were all greater than grade II. Patients with secondary cataracts due to trauma, toxins, inflammation, and degenerative ocular diseases were excluded from the study. In addition, patients with diabetes, hypertension, high myopia, glaucoma, any syndrome and those who were smokers, alcoholics, had exposure to UVB radiation, or took medication such as steroids were also not considered. A total of 156 unrelated healthy individuals from the same ethnic background without a history of cataracts served as normal controls. The control subjects were selected randomly during a routine medical fitness examination, which included ophthalmic examination.

Informed consents were obtained from all subjects after explaining the nature and possible consequences of the study. All experiments were approved by the Institutional Review Board of Harbin Medical University (Harbin, China) and were conducted according to the principles in the Declaration of Helsinki.

### DNA analysis

Five milliliters samples of venous blood were collected in EDTA vaccutainers (BD, San Jose, CA) from ARC patients and control subjects. Genome DNA was extracted from peripheral blood leukocytes using the QIAamp DNA Blood Mini Kits (QIAGEN Science, Germantown, MD). The primers (Table 1) for polymerase chain reaction (PCR) were designed using Primer3 according to the reference sequences in the NCBI Gene database. We sequenced the PCR products with an ABI3730 Automated Sequencer (PE Biosystems, Foster City, CA), and analyzed the sequencing results using Lasergene SeqMan (DNASTAR, Madison, WI).

**Table 1:** Primers used for polymerase chain reaction (PCR) in this study.

**Primer name**	**Forward (5′-3′)**	**Reverse (5′-3′)**
GJA3–1	TGCCCATCAGCCCCATCCCAGTA	AGCCCACCTCGAACAGCGTCTTGA
GJA3–2	CTACCTGGGCCACGTGCTGC	GCTTGGCGGACTGGCCCTTT
GJA3–3	TCGGGTTCCCACCCTACTAT	TATCTGCTGGTGGGAAGTGC
GJA8–1	CCGCGTTAGCAAAAACAGAT	GCTGCTCTACAGGCCTCTTC
GJA8–2	TGGCCTCTGTGTCCCTATTC	GTTGGCACCTTTTCCTTTCA
LIM2–1	CATCTCCTTCTCCCAAGCAC	ACCTCTGAAGCGTCAGGAAA
LIM2–2	GTGGTGGGGGTGTTTATGAC	GGGTTGAGTGTGAGGAGGAG
LIM2–3	CACCCCTTTCCCCAATCTTA	CACAAACCCACAGTCCAGAA

### Simulation for functional change in coding nonsynonymous SNPs (nsSNPs)

PolyPhen online software, which is based on the position-specific independent counts (PSIC) score derived from multiple sequence alignments of observations [20], was used to investigate the possible impact of all nonsynonymous changes on the structure and function of GJA3, GJA8, and LIM2. PolyPhen scores of >2.0 indicate that the variation is likely damaging to protein function; scores of 0.5–2.0 are possibly damaging; and scores of <0.5 are likely benign.

### Cross species conservation analysis

We downloaded the amino acid sequences from the NCBI HomoloGene database and evaluated the cross species conservation of the nonsynonymous mutations using Lasergene MegAlign (DNASTAR).

## Results

### Characteristics of patients

A total of 301 unrelated subjects were recruited in this study. The cohort consisted of 145 unrelated cases with age-related cataracts and 156 random control subjects. The mean age of the cases was 70.77±8.46 and that of controls was 50.72±11.82.

### Identification of novel mutations

**Figure 1:**
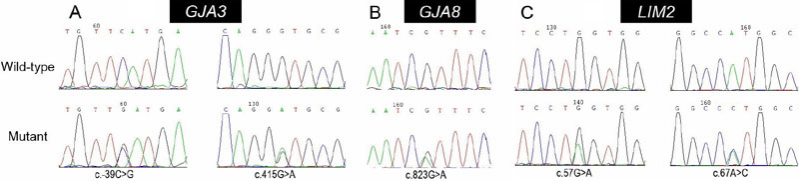
The sequence chromatograms (forward strand). **A**: The variations (c.-39C>G, c.415G>A) we found in gap junction protein α3 (*GJA3*). **B**: A mutation (c.823G>A) in gap junction protein α8 (*GJA8*). **C**: Two variations (c.57G>A, c.67A>C) in lens intrinsic membrane protein 2 (*LIM2*).

We found five novel sequence changes in 145 patients and none of them presented in the 156 controls. In *GJA3*, we found two variations c. −39C>G and c. 415G>A lying in the 5′ UTR and the exon, respectively (Figure 1A). Among them, the nonsynonymous change c. 415G>A leads to p.V139M. In *GJA8*, a 70-year-old woman with cortical cataracts was found to carry a heterozygous missense mutation c. 823G>A (Figure 1B) that leads to p.V275I. In *LIM2*, a 56-year-old women with cortical cataracts was found to carry a heterozygous sequence alteration c.67A>C that leads to p.M23L; meanwhile, another 62-year-old woman carried a heterozygous synonymous mutation c.57G>A (Figure 1C). There was no other mutation in these three candidate genes.

The number, gender, age, and clinical type (both eyes) of patients who carried genetic variations were listed in Table 2. After the laboratory work, we paid a return visit to the mutation carrying patients and confirmed they had no family history of cataracts.

**Table 2:** Clinical data of patients who carried genetic variation.

**Serial number**	**Gender**	**Age**	**Type (both eyes)**	**Gene**	**Variation**
19	Female	70	Cortical	*GJA8*	c.823G>A
66	Female	75	Nuclear	*GJA3*	c.-39C>G
78	Female	56	Cortical	*LIM2*	c.67A>C
79	Male	76	Cortical	*GJA3*	c.415G>A
99	Female	62	Cortical	*LIM2*	c.57G>A

*GJA3*, gap junction protein α3; *GJA8*, gap junction protein α8; *LIM2*, lens intrinsic membrane protein 2.

### Effects of nonsynonymous changes and cross species conservation by in silico analysis

Using PolyPhen, p.M23L in LIM2 is predicted to be possibly damaging, while p.V139M in GJA3 and p.V275I in GJA8 are predicted to be benign. The scores and results of PolyPhen are listed in Table 3.

**Table 3:** Variations found in GJA3, GJA8, LIM2 and PolyPhen results of the nonsynonymous mutations.

**Gene**	**Nucleotide notation**	**Protein notation**	**Polyphen score**	**Polyphen prediction**
*GJA3*	c.-39C>G	-	-	-
	c.415G>A	p.V139M	0.058	Benign
*GJA8*	c.823G>A	p. V275I	0.252	Benign
*LIM2*	c.57G>A	synonymous	-	-
	c.67A>C	p.M23L	1.595	Possibly damaging

*GJA3*, gap junction protein α3; *GJA8*, gap junction protein α8; *LIM2*, lens intrinsic membrane protein 2.

**Figure 2:**
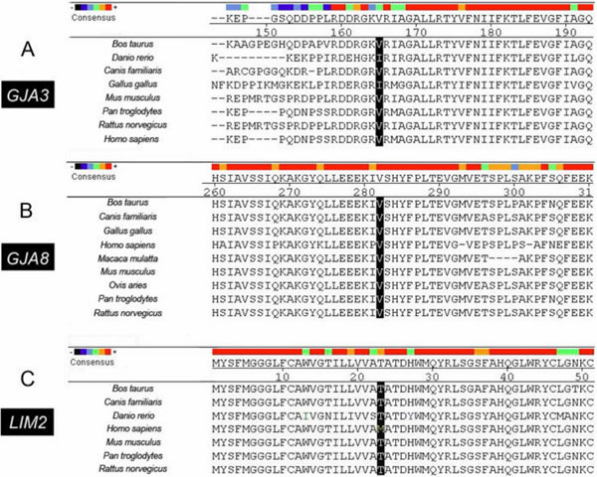
Cross species conservation analysis. The black bars highlight the interesting positions of the proteins. **A**: Multiple alignment indicates that valine at position 139 in gap junction protein α3 (GJA3) is medium conserved. **B**: Valine at codon 275 in gap junction protein α8 (GJA8) is highly conserved. **C**: At lens intrinsic membrane protein 2 (LIM2) position 23, methionine was not a conserved amino acid residue compared to threonine.

We aligned the amino acid sequences of GJA3, GJA8, and LIM2 from several species. The valine at codon 139 in GJA3 is medium conserved (Figure 2A) and the valine at position 275 in GJA8 is highly conserved (Figure 2B). In LIM2, tyrosine is more conservative in other species compared with methionine at codon 23 in human (Figure 2C).

## Discussion

In general, maintenance of an intact, transparent lens requires the balanced homeostasis of metabolic components [21]. The proteins on the membrane of lens fiber cells, such as connexins, are crucial for the maintenance of the homeostasis in the whole lens.

*GJA3* and *GJA8* encode gap junction protein α3 (connexin 46) and α8 (connexin 50), which express on the lens fiber cell membrane. Gap junctions construct an extensive network, which is vital for the maintenance of osmotic and metabolic balance in the avascular lens [22]. Defects in *GJA3* and *GJA8* have previously been reported to cause cataracts in humans and mice [23,24] whereas previous studies of congenital cataract associated with connexin 46 did not find any change in the intracellular loop (CL) of the protein. In this study, we found a 76-year-old man with cortical cataracts carried a heterozygous mutation c. 415G>A that lead to p.V139M lying in the middle of CL. It is the first report about an amino acid residue change in the intracellular loop of connexin 46. However, we still need more experiments to figure out how this mutation affects cataract formation.

In *GJA8*, the mutation we detect is located in the COOH-terminus. Now, few mutations in connexin genes that have been reported to be associated with cataracts lay in the COOH-terminus. Indeed, removing the COOH-terminus (139–150 amino acids) of connexin 50 did not inhibit the formation of homotypic or heterotypic channels, it only caused a loss of pHi sensitivity and a decrease in conductance [25,26]. In 2008, Schmidt et al. [27] found a homozygous insertion of one G after position 776 of *GJA8*, leading to a frame shift and 123 novel amino acids, causing a recessive triangular cataracts in a German family. Polyakov et al. [28] found a mutation (I274M) in a Russian family with zonular pulverulent cataracts in 2001, but in 2009 this allele was thought to be a rare polymorphism, not a cataract-causing mutation [29]. Yan et al. [30] found a mutation (S276F) causing a dominant congenital pulverulent nuclear cataract in a Chinese family in 2008. These findings suggested that mutation in the COOH-terminus of connexin 50 may somehow interfere with the normal function of the gap junction and lead to cataract. The mutation V275I we found in the present study is between I274M and S276F in the COOH-terminus of connexin 50. The high conservation of valine at codon 275 from zebrafish to humans (Figure 2B) indicates the importance of this residue.

The product of *LIM2* is a 173 amino acids membrane protein, with four intramembrane domains [31], expressed mainly in the cortical region of the lens [32]. The function of this protein remains unknown. Taylor et al. [33] suggested that it has an important role in the switch from proliferation to differentiation and in the maintenance of the cells in the differentiated state. Mutations in *LIM2* were related to autosomal recessive congenital cataracts in humans. Simultaneously, the heterozygous *To3* mice developed congenital total cataracts, but in the homozygous state they also appear microphthalmia [34]. The semidominant pattern of the *To3* mutation highlights the genetic complexity of *LIM2* in the formulation of cataracts [35]. In our study, the synonymous and nonsynonymous mutations were found in both female patients with cortical cataracts; their ages were 62 and 56 years old. The nonsynonymous mutation (c.67A>C) that leads to the alteration of methionine to leucine at codon 23 of *LIM2* was predicted as potentially damaging to the protein’s function by PolyPhen, although the methionine was not a conserved amino acid residue among species (Figure 2C).

On one hand, the mutations in the coding region may affect the normal function of the protein more obviously; on the other hand, the variations in untranslated region are also important to the translational regulation [36,37]. Disturbance of the regulation of the translational machinery leads to perturbed cellular metabolism and may shift the physiologic balance from healthy to diseased states, such as breast cancer, Alzheimer disease, bipolar affective disorder, fragile X-syndrome, or others [38]. Meanwhile, synonymous changes may also contribute to the development of human diseases [39]. As such, the variation c. −39C>G in the 5′UTR of *GJA3* and the synonymous mutation c.57G>A in *LIM2* may affect the normal process of translation. Unfortunately, due to a lack of lens tissues, we could not examine the effect of these mutations on the RNA level.

Nonetheless, ARC is considered a multi-factorial disease, where environmental components as well as genetic predisposition contribute to the development of the pathological condition. This study revealed a slight association between the three lens fiber cell membrane proteins genes (*GJA3*, *GJA8*, and *LIM2*) and age-related cataract development in the Chinese population. We need further studies to find out the precise mechanisms by which genetic mutations of these genes influence the natural history of age-related cataract development.
